# Metformin-associated lactic acidosis presenting as an ischemic gut in a patient who then survived a cardiac arrest: a case report

**DOI:** 10.1186/1752-1947-8-159

**Published:** 2014-05-21

**Authors:** Dumisani Ncomanzi, Rhea Mae R Sicat, Krishnaswamy Sundararajan

**Affiliations:** 1 Intensive care unit, Critical care services, Level 4, Robert Gerard Wing, Royal Adelaide Hospital, North terrace, Adelaide, SA 5000, Australia; 2 Senior Clinical Lecturer, Discipline of Acute care Medicine, University of Adelaide, Adelaide, SA 5000, Australia

**Keywords:** Cardiac arrest, Gut, Ischemia, Lactic acidosis, Metformin

## Abstract

**Introduction:**

Lactic acidosis is the most common cause of metabolic acidosis in hospitalized patients. It is recognized as a potential complication of metformin use, particularly in patients with risk factors such as renal dysfunction, liver disease, and heavy alcohol ingestion. These conditions are associated with systemic hypoxemia, which may be caused by cardiorespiratory disease, major surgery, sepsis, dehydration, old age, and overdose. The reported frequency of lactic acidosis is 0.06 per 1000 patient-years, mostly in patients with predisposing factors. This case is important because it details the seriousness of metformin-associated lactic acidosis in a critically ill patient and because, to the best of our knowledge, our patient survived with minimal residual defect despite experiencing a cardiac arrest.

**Case presentation:**

A 66-year-old Caucasian woman presented to our hospital with profound lactic acidosis, which was initially thought to be ischemic gut. She then survived an in-hospital pulseless electrical activity arrest.

**Conclusion:**

Metformin-associated lactic acidosis is a diagnosis by exclusion; however, a high degree of clinical suspicion supplemented by prompt multisystem organ support can significantly influence the outcome in critically ill patients.

## Introduction

Metformin is an oral biguanide anti-diabetic agent. It is a small, non-plasma-protein-bound molecule, and 90% of metformin is excreted unchanged by the kidneys through glomerular filtration and possibly through tubular secretion. In addition to being relatively safe, its use has been advocated in the treatment of type 2 diabetes in patients who are obese and has been shown to slow cardiovascular complications associated with diabetes. By decreasing excess hepatic gluconeogenesis without raising insulin levels, it rarely leads to significant hypoglycemia when used as monotherapy [1-3]. Therapy with metformin has been associated with type B lactic acidosis.

Metformin-associated lactic acidosis (MALA) is a grave but infrequent complication of metformin use and reportedly has a mortality rate up to 50%. There has been a serious debate about the exact role of metformin in the development of lactic acidosis. The Cochrane group [4], Comparative Outcomes Study of Metformin Intervention versus Conventional Approach (COSMIC) [5] study, and the United Kingdom Prospective Diabetes study [6] have disputed the existence of lactic acidosis in the presence of metformin and hence the term metformin-induced lactic acidosis has subsequently been changed to MALA. The independent effect of metformin on the development of lactic acidosis remains unclear. Metformin reduces pyruvate dehydrogenase activity and mitochondrial transport of reducing agents, and thus enhances anaerobic metabolism. This shift to anaerobic metabolism, in the presence of reduced insulin, increases production of precursors for the Krebs cycle. Consequently, there is a decreased ability to channel those precursors into aerobic metabolism, which, in turn, results in increased metabolism of pyruvate to lactate and increases net lactic acid production. MALA has non-specific presenting features; typically, patients have severe hypotension with low systemic vascular resistance and respiratory failure.

A systematic review and meta-analysis showed no evidence that metformin therapy is associated with an increased risk of lactic acidosis or with increased levels of lactate compared with other anti-hyperglycemic treatments if the drug is prescribed under study conditions, taking into account contraindications [7]. Of 194 studies considered, there were no cases of fatal or nonfatal lactic acidosis in 36,893 patient-years in the metformin group or in 30,109 patient-years in the non-metformin group [7]. In another review of 11,800 patients treated with metformin for a mean of about two years in Saskatchewan, Canada, only two patients developed lactic acidosis (incidence: nine cases per 100,000 years of exposure) [8].

## Case presentation

A 66-year-old Caucasian woman with type 2 diabetes presented to our emergency department with a three-week history of generalized malaise, associated poor oral intake, and some diarrhea. Her enteric symptoms were vague and unquantifiable. She was obese and had a past medical history of poorly controlled type 2 diabetes for 15 years, hypertension, asthma, and depression. Her regular medication comprised metformin 3g daily, modified-release gliclazide 60mg daily, aspirin 100mg daily, atorvastatin 40mg daily, ramipril 10mg daily, and hydrochlorothiazide 25mg daily. Our patient was brought into hospital by ambulance; her pre-hospital observations were as follows: Glasgow Coma Scale score, 15; blood sugar level, 2.8mmol/L; blood pressure, 90/40mmHg; pulse, 54 beats per minute; respiratory rate, 32 breaths per minute; and peripheral oxygen saturation, 98% on 8L oxygen via a variable oxygen delivery mask. On arrival to our emergency department, she was confused with a Glasgow Coma Scale score of 14 out of 15 (E4V4M6), with the rest of her physiological parameters similar to her pre-hospital observations.

Her initial investigations were as follows: serum sodium, 140mmol/L (normal range: 137 to 145mmol/L); serum potassium, 7.3mmol/L (normal range: 3.5 to 4.9mmol/L); serum chloride, 91mmol/L (normal range: 100 to 109mmol/L); serum bicarbonate, 1mmol/L (normal range: 22 to 32mmol/L); anion gap, 55mmol/L (normal range: 7 to 17mmol/L); serum glucose, 2.3mmol/L; urea, 30.8mmol/L (normal range: 2.7 to 8.0mmol/L); and serum creatinine, 768umol/L (normal range: 50 to 100umol/L). Results from liver function tests were normal. Her troponin level was 50ng/L (normal range: <30ng/L). Venous blood gas measurements revealed a profound metabolic acidemia: pH, 6.58; partial pressure of CO_2_, 38.6mmHg; HCO_3_, 3.6mmol/L; glucose, 2.0mmol/L; and lactate, 16.7mmol/L. Her initial resuscitation strategy included 2000mL of 0.9% sodium chloride solution, 10mL of 10% calcium gluconate, 15 units insulin (Actrapid) in 50mL 50% dextrose, and 1mL/kg of 8.4% sodium bicarbonate solution.

Minutes after these initial investigations, our patient experienced a pulseless electrical activity cardiac arrest and was managed as per advanced life support protocol. She had a total downtime of 25 minutes. During cardiopulmonary resuscitation she was intubated and ventilated. Following return of spontaneous circulation, she required an infusion of adrenaline for blood pressure support. She had an unremarkable chest radiograph, electrocardiogram, and toxicology screen.

With a presumed diagnosis of ischemic bowel based on vague abdominal features and profound lactatemia, our patient was admitted to our intensive care unit (ICU) for preoperative optimization. In our ICU, she was sedated and ventilated on an inspired oxygen concentration of 40% and on modest ventilator paramters (peak inspiratory pressure <25cmH_2_O). To treat her severe circulatory shock, she was fluid-resuscitated with a total of 10,000mL crystalloid from a central venous pressure of 8cmH_2_O to 16cmH_2_O. In addition, she required very high doses of noradrenaline and adrenaline. Continuous veno-venous hemodiafiltration was commenced at exchange rates of 50mL/kg/h using Hemosol B0 solution. She was empirically started on vancomycin and piperacillin and tazobactam (Tazocin) as broad-spectrum antimicrobial cover.

Our patient went to theater 26 hours post admission for an exploratory laparotomy, which revealed no significant findings. We continued her broad-spectrum antimicrobial cover despite negative microbiological cultures. Renal replacement therapy continued and our patient’s acid-base balance slowly normalized over three days. Having excluded all causes of a high anion gap lactic acidosis, including negative red blood cell transketolase activity for thiamine deficiency, we presumed our patient to have had a severe MALA ‘triggered’ by an acute kidney injury from dehydration. This was supported by a serum metformin level of 4mg/L. Her renal function slowly improved with continuous veno-venous hemodiafiltration and she slowly recovered with a total of 35 days stay in ICU.

## Discussion

A high anion gap metabolic acidosis commonly occurs secondary to ketoacidosis (in diabetes, alcoholism, starvation), toxins (alcohol, ethylene, glycol, methanol, and paraldehyde), kidney injury, and lactic acidosis (shock, hypoxia, carbon monoxide, cyanide, and metformin). A diagnosis of MALA should always form part of the differential in a patient with high anion gap metabolic acidosis. Even though MALA masquerading as an ischemic gut has previously been reported, our case is different because our patient survived, as compared to the previous report in which the diagnosis was made posthumously [9].

Treatment of MALA is largely supportive, comprising adequate resuscitation, treating any underlying confounding disease process, and renal replacement therapy for acidosis and drug elimination. Lactic acidosis, though extremely rare, is associated with 50% mortality. An analysis of 49 cases of lactic acidosis associated with metformin use found the overall mortality was not correlated with plasma lactate concentrations. Paradoxically, plasma metformin concentrations were about three times higher in patients who survived. All cases of lactic acidosis had, in addition to metformin use, acute or chronic comorbidities predisposing to lactic acidosis, suggesting that lactic acidosis may be coincidental rather than causally associated with metformin use [10]. Another case series concluded that the extent of acidosis and accumulation of metformin was less important than other coexisting factors [11]. A good predictive factor of death is an acute liver dysfunction as assessed by prothrombin time.

Our patient had multiple risk factors, including renovascular disease, an acute kidney injury from dehydration, and continued intake of nephrotoxic angiotensin-converting enzyme inhibitor and thiazide diuretic. In addition, she had continued taking her usual metformin dose. We believe that there was an accumulation of metformin due to reduced renal clearance. Her severe lactic acidosis with increasing inotrope requirement while in our ICU led to an emergency laparotomy, which did not show any intra-abdominal pathology. Her plasma metformin level subsequently came back as 4mg/L, which is suggestive of our diagnosis in the absence of any other cause in a patient presenting with a raised anion gap (44.6mmol/L) metabolic acidosis. It is worth noting that the incidence of MALA estimated from metformin serum concentration measurements in patients with type 2 diabetes mellitus is 5 to 16 times higher than reported in the literature [11].

Our case typifies MALA in that there were high serum metformin levels, profound lactatemia in the presence of an Acute kidney injury, and concurrent illness. In this regard, is metformin causal, co-responsible, or coincidental? The debate rages on.

## Conclusion

MALA is rare but life threatening. Although controversy surrounding causality exists, current guidelines advocate metformin review and/or withdrawal for patients with renal impairment, during periods of tissue hypoxia, two days before general anesthesia, and for three days following use of contrast-medium-containing iodine. Early renal replacement therapy forms the mainstay of treatment in critical illness because it provides both symptomatic and etiological treatment by eliminating lactate and metformin, in addition to cardiorespiratory organ support. Goals of supportive treatment are to maintain adequate tissue perfusion, correct electrolyte imbalance and hypoglycemia, and perform mechanical ventilation in patients with respiratory distress and hemodynamic instability.

## Consent

Written informed consent was initially obtained from the patient’s next of kin as the patient was too unwell to sign a valid consent form at the time of obtaining the consent. Subsequently, written informed consent was also obtained from the patient as she had recovered completely and was deemed to be mentally competent to give her consent for her case to be published. A copy of the written consent is available for review by the Editor-in-Chief of this journal.

## Abbreviations

ICU: intensive care unit; MALA: metformin-associated lactic acidosis.

## Competing interests

The authors declare that they have no competing interests

## Authors’ contributions

DN obtained background information about the patient, performed the literature search and prepared the manuscript. RS assisted with literature search, writing the manuscript and providing information about the patient. KS obtained consent from the patient and family and supervised the work along with editing the manuscript and offering intellectual input and technical expertise in finalizing the manuscript. All authors have read and approved the final manuscript.
